# Plastic Waste Degradation in Landfill Conditions: The Problem with Microplastics, and Their Direct and Indirect Environmental Effects

**DOI:** 10.3390/ijerph192013223

**Published:** 2022-10-14

**Authors:** Irena Wojnowska-Baryła, Katarzyna Bernat, Magdalena Zaborowska

**Affiliations:** Department of Environmental Biotechnology, University of Warmia and Mazury in Olsztyn, Sloneczna 45G, 10-709 Olsztyn, Poland

**Keywords:** microplastics (MPs), plastic breakdown and degradation, landfill leachate, landfill gases, pollutant vectors and carriers

## Abstract

As landfilling is a common method for utilizing plastic waste at its end-of-life, it is important to present knowledge about the environmental and technical complications encountered during plastic disposal, and the formation and spread of microplastics (MPs) from landfills, to better understand the direct and indirect effects of MPs on pollution. Plastic waste around active and former landfills remains a source of MPs. The landfill output consists of leachate and gases created by combined biological, chemical, and physical processes. Thus, small particles and/or fibers, including MPs, are transported to the surroundings by air and by leachate. In this study, a special focus was given to the potential for the migration and release of toxic substances as the aging of plastic debris leads to the release of harmful volatile organic compounds via oxidative photodegradation. MPs are generally seen as the key vehicles and accumulators of non-biodegradable pollutants. Because of their small size, MPs are quickly transported over long distances throughout their surroundings. With large specific surface areas, they have the ability to absorb pollutants, and plastic monomers and additives can be leached out of MPs; thus, they can act as both vectors and carriers of pollutants in the environment.

## 1. Introduction

Microplastics (MPs) pollution is released to the environment directly from primary sources (such as cosmetic and cleansing commodities, and personal care and pharmaceutical products), or indirectly from secondary sources, through the fragmentation and degradation of nano-, meso- and macroplastics [1]. The identified primary sources of MPs emissions to the environment include plastic pellets from industry, washing machines, which release a large amount of MPs in the form of fibers [2], microbeads from personal care products (PCPs) and paint, wastewater treatment plants (WWTPs), rubber roads, artificial turf, and tire wear [3]. Emissions from WWTPs are considered to be one of the main sources of MPs released to the environment as large amounts of effluent are directly discharged into surface water every year. Conley et al. [4] found that MPs concentrations (counts per L) varied within a factor of 2.5 in influent and 4.8 in effluent from each of three monitored WWTPs, and that neither concentrations nor removal efficiencies demonstrated a seasonal trend. The WWTPs that operated with primary clarification had the highest MPs removal efficiency of 97.6 ± 1.2%. The two smaller facilities had average removal efficiencies of 85.2 ± 6.0% and 85.5 ± 9.1%. Based on flow rates and MPs counts, the load of MPs discharged with the effluent totaled 500–1000 million MPs particles per day. This is equivalent to emissions of 0.34–0.68 g MPs per capita per year in treated wastewater. In addition, because activated sludge also accumulates most of the removed MPs (69–80%), it can be an emission source if improperly managed [5].

Another source of microplastics emissions is larger plastic products that are not properly disposed of, mainly including plastic films from agricultural applications, fishing waste, and municipal debris from plastic bags, bottles, tableware, and packing products [6]. Landfills are major reservoirs of MPs, including tiny plastic granules used in cosmetics and small plastic fragments derived from the breakdown of macroplastics. Currently, secondary sources are estimated to emit the majority of MPs to the environment even though, under natural conditions, it takes years for large plastic waste to be broken down into MPs [3]. For example, microplastic films and foams are derived mainly from the erosion of plastic bags and packing products, which are essential items in humans’ daily lives [7]. Since the 1990s, they have been widely used because of their advantages of a low cost, large capacity, light weight, and easy storage. Globally, up to ∼five trillion plastic bags are consumed every year and ∼39.7% of total plastic production is used for packing [8].

Most of this plastic waste is landfilled and is subject to long-term degradation. Plastic materials undergo significant changes in their chemical structure under specific environmental conditions, resulting in the loss of some of their properties. Polymeric characteristics play a significant role in the degradation. These characteristics include molecular weight, crystallinity, functional groups, mobility, substituents present in the structure, and the additives added to the polymers. The fate of these polymers in the landfill and the time required for their total mineralization to CO_2_ have yet to be fully understood. Polymers can be degraded via chemical degradation, photodegradation, and biological degradation, which may lead to the formation of secondary MPs pollution.

The presence of MPs in the environment has been shown to have numerous hazardous effects on a wide range of floral and faunal species. In addition, MPs act as carriers or chelators of various co-contaminants, such as heavy metals, brominated flame retardants and other types of plasticizers, and pharmaceutical toxicants. These co-contaminants easily bind to the microplastic surface due to their hydrophobicity [9]. The microbial species that have been associated with the degradation of these polymers include bacteria (*Pseudomonas*, *Streptococcus*, *Staphylococcus*, *Micrococcus*) and fungi (*Aspergillus niger*, *Aspergillus glaucus*, *Trichoderma*) [10]. The extracellular enzymes involved are too large to penetrate deeply into the polymer material, acting only on the polymer surface, and consequently, the biodegradation of plastics is usually a surface erosion process. Biodegradation can be improved by UV irradiation (photooxidation), and thermal and chemical oxidation of polyethylene (PE) prior to its exposure to a biotic environment.

‘End-of-life’ (EoL) does not equate to the end of the impact of plastic waste. Plastic materials persist and pollute long after their intended use is finished, which means that there is no such thing as an end to the impact of plastics within a typical human lifespan. Depending on how plastic is handled, it may pose a significant threat to the environment and to the climate when it reaches the waste phase of its life cycle.

The objective of this article was to delineate the complications—both environmental and technical—encountered during the disposal of plastic products when they are landfilled at their EoL. A special focus was given to the potential for the migration and release of various types of microplastics. In addition, the emissions of potentially toxic substances during landfilling were assessed.

## 2. Plastic Waste Generation and Disposal

Plastics in the global ecosystem are distributed between three groups: plastics in use, post-consumer managed plastic waste, and mismanaged plastic waste (MPW), which includes urban litter. Plastic waste is dominated by thermoplastic types of polypropylene (PP) (21%), low-density and linear low-density polyethylene (LDPE and LLDPE) (18%), polyvinyl chloride (PVC) (17%), and high-density polyethylene (HDPE) (15%). Other plastic types include polystyrene (PS) and expandable PS (EPS) (8%), polyethylene terephthalate (PET) (7%, excluding PET fibers), and thermosetting plastic polyurethane. The types of MPs that are generated and released vary with the composition of the plastic waste from which they are derived, and are identified and synthesized using dispersed data on the production, use, and end-of-life management of polymer resins, synthetic fibers, and additives. Geyer et al. [11] presented a global analysis of all mass-produced plastics ever manufactured. A total of 8300 million metric tons (Mt) of virgin plastics has been produced to date. As of 2015, approximately 6300 Mt of plastic waste has been generated, around 9% of which has been recycled, 12% has been incinerated, and 79% has accumulated in landfills or the natural environment. If current production and waste management trends continue, roughly 12,000 Mt of plastic waste will be in landfills or in the natural environment by 2050.

In 2018, landfills in the European Union (EU) received 7.2 million tons of plastic. Packaging plastics made of HDPE, LDPE, PP, PET, PS, and PVC constitute a significant portion of all plastics in landfills [12]. The total consumption of LDPE, HDPE, PP, PS, EPS, PVC, and PET was 90.0 ± 4.8 kg/cap in 2016. The in-use stock that year was 47 kg/cap for LDPE, 81 kg/cap for HDPE, 81 kg/cap for PP, 21 kg/cap for PS, 56 kg/cap for EPS, 163 kg/cap for PVC, and 16 kg/cap for PET. For EPS and PVC, the fraction contained in the in-use stock was 51% and 39% of the total production. Landfills constitute the major repository of LDPE, HDPE, PP, PS, and PET (48–60%) [13].

A landfill is an ecological system, where the inputs are solid waste and water, and the outputs are leachate and gas produced by the joint action of biological, chemical, and physical processes. The typical municipal solid waste (MSW) landfill conducts five phases of waste treatment: (1) initial adjustment (aerobic biodegradation); (2) transition (from aerobic conditions to anaerobic conditions); (3) acid formation (hydrolysis); (4) methane fermentation (methanogenesis); and (5) final maturation and stabilization of the solid waste. Once landfilled, plastics undergo aerobic biodegradation (at an initial stage), but soon make the transition to anaerobic conditions, with the consequent influences on plastic degradation of acid formation and methane fermentation of organic solid wastes. Even in the absence of light and oxygen, landfilled MPs will continue to fragment into nanoplastics due to the fluctuating temperatures (reaching as high as 60 to 90 °C) and pH (4.5–9), deep-seated fires, physical stress, and compaction, as well as limited microbial activity.

Each phase accelerates the breakdown and degradation of plastics. Most of the polymers and plastics in landfills remain unchanged, or they may degrade via some biotic or abiotic process into fragments that either remain as produced or biodegrade to gaseous products and water. The gaseous products are carbon dioxide in aerobic environments and a mixture of carbon dioxide, methane, and volatile organic compounds (VOCs) in an anaerobic environment.

The ultimate fate of plastic in landfills is a major concern, particularly as there is no established method of determining whether the plastic degrades, biodegrades, or is recalcitrant. Degradation or biodegradation may have potential negative effects such as the destabilization of the structural stability of the landfill.

Landfills are designed to prevent any exchange with the surrounding environment. Leachate recycling landfills are designed to capture and recycle aqueous leachate to prevent or reduce the environmental leakage of potentially harmful waste or degradation residues. Controlled contaminant release landfills allow for the gradual migration of leachate into the environment under closely monitored conditions to ensure that no harmful events happen. Unrestricted contaminant release landfills, which are older waste dumps, have no controls on leachate or environmental contamination. Like the variation in plastic waste inputs, the types of MPs generated and released will vary with the composition of different MP polymers. Near an abandoned coastal landfill, PS, PET, PA, and PP were the dominant MP polymers [14].

A large amount of plastics is buried in landfills. As landfills are relatively closely sealed reactors with complex biochemical reactions and physical changes, plastic waste buried in landfills is subjected to much more severe environmental conditions, e.g., leachate pH (varying from 4.5 to 9), high salinity, fluctuating temperature, gas generation (e.g., CO_2_ and CH_4_), physical stress, and microbial degradation. All of the above factors may lead to the fragmentation of plastics to MPs, and small plastic debris could be carried out by the discharge of leachate.

The abundance of MPs in landfill refuse is 20,000–91,000 items/kg, which is much higher than the concentrations in sediments, sewage sludge, and agricultural soil [15]. Su et al. [16] suggested that landfills were not only the primary disposal sites of plastic waste, but also important sources of MPs. They found that the average abundance of MPs in refuse was 62 (±23) items/g. Fragments and PE were the predominant shapes and polymer type, respectively, of the MPs in refuse. The authors identified irregular shapes and hack structures in refuse microplastics, which indicates that the breakdown of plastic debris was the primary contributor to MPs formation, specifically secondary MPs in landfills. The microplastics in landfill leachate originated mainly from the breakdown of different kinds of plastic products, and thus the proportion of plastic fibers is influenced by the amount of plastic fiber products that are landfilled [11].

It is possible to assess the degree of degradation of plastics mined from a landfill based on their characteristics. According to Zhou et al. [17], after 24 years of landfilling, plastic wastes accounted for 10.62 ± 5.12% of the total stored wastes in an old landfill. Of these plastic wastes, 69.13% were plastic bags 11.34% were white PE plastic bags; 29.77% were colored PE plastic bags; 28.02% were other plastic bags; and 30.87% were other plastics, including PP, PVC, and PS. Compared to normal plastic wastes, the landfill plastic waste had a significantly lower content of carbon and sulfur, but a higher content of oxygen, silicon, aluminum, and a similar content of nitrogen and chlorine. The ash content of landfill-mined plastic waste was 9.03 times higher than that of normal plastic waste. Surface images of normal plastic waste and the landfill plastic waste indicated that there were many particles that had heavier molecular weights than normal plastic, with particle sizes ranging from 5 to 60 mm.

The application of suitable technology for the management of waste landfills is necessary for reducing the volume of N/MPs in the environment. A semi-aerobic landfill is a concept for solid waste disposal which accelerates waste decomposition with low emissions of methane. Air is supplied into these landfills through leachate collection pipes by heat convection between the higher-temperature waste and the lower-temperature atmosphere. In semi-aerobic landfill conditions, the oxygen content in the waste bed varied between 3% and 8% (*v*/*v*), and the highest temperature in the landfill chamber was 75.8 °C [18]. Although many microorganisms in waste play important roles in degrading persistent wastes, methanotrophs are particularly important for their roles in reducing global warming and natural resource contamination. These microorganisms utilize methane as their sole carbon and energy source. Over 300 persistent compounds, such as aliphatics, alkanes, aromatics, and chlorinated hydrocarbons, are co-metabolized by the methane monooxygenase (MMO) enzyme produced by methanotrophs [19]. According to Muenmee et al. [20], methane oxidation rates correlated with percent losses of plastics, indicating the importance of methanotrophs for plastic degradation. The biodegradation of plastic wastes in a semi-aerobic landfill could be accelerated by supplying aeration in proportion to the methane available in the waste bed. An available oxygen concentration of about 3–8% *v/v* accelerated the biodegradation of plastic waste and significantly increased weight percent losses (15–20% of HDPE and 5–9% of PP). The use of a semi-aerobic landfill for MSW not only reduced methane emissions, but also improved plastic waste deterioration. Ishigaki et al. [21] applied forced aeration to a landfill reactor to create aerobic conditions that could potentially stimulate polymer-degrading microorganisms. The individual degradation behaviour of biodegradable plastics (BPs) varied widely under aerobic conditions. Polycaprolactone plastic, a chemically synthesized BP, showed film breakage under both aerobic and anaerobic conditions, which may have contributed to a reduction in the volume of this waste regardless of the variant. Polyhydroxybutyrate and hydroxyvalerate plastics were effectively degraded in aerobic conditions but not in anaerobic conditions. In contrast, aeration did little to accelerate the volume reduction of a blend of starch and polyvinyl alcohol (SPVA) plastic and cellulose acetate plastic. The degradation of these BPs may have been prevented by the recalcitrant portions of the plastics, such as the polyvinyl alcohol and the highly substituted cellulose acetate. The great variability in the degradability of BPs in aerobic and anaerobic waste landfills suggests that landfilling must be combined with the utilization of BPs to improve the reduction of waste volume in landfill sites.

## 3. Bio-/Degradation of Petroleum-Based Plastic

In the case of petroleum-based plastic, degradation is often synonymous with biodegradation. Degradation is any physical or chemical change in a polymer that results from environmental factors, such as light, heat, moisture, chemical conditions, or biological activity. These changes include bond scission, chemical transformation, and the formation of new functional groups [22]. Degradation is reflected by changes in material properties, such as mechanical, optical, or electrical characteristics, and by crazing, cracking, erosion, discoloration, phase separation, or delamination.

In photodegradable plastics, the groups that are connected to the polymer backbone are light-sensitive. When they are exposed to UV radiation for a long time, their polymeric structure can be disintegrated. However, when radiation exposure is stopped, degradation is not possible. As the interiors of landfills lack sunlight, plastics in landfills degrade via different means. Artificial photodegradation can be problematic, as it may lead to the release of toxic VOCs, which are potentially hazardous and associated with the environmental weathering of plastic debris [23]. The susceptibility of polymers to photodegradation is related to their ability to absorb the harmful part of tropospheric solar radiation. This includes UV-B radiation (~295–315 nm) and UV-A radiation (~315–400 nm), which are responsible for direct photodegradation (photolysis, initiated photooxidation). The visible part of sunlight (400–760 nm) accelerates polymeric degradation by heating, and infrared radiation (760–2500 nm) accelerates thermal oxidation [22]. Most plastics tend to absorb high-energy radiation in the ultraviolet portion of the spectrum, which increases the reactivity of their electrons and causes oxidation, cleavage, and other degradation [24].

The degradation of most synthetic plastics is a very slow process that involves environmental factors, followed by the action of microorganisms. The primary mechanism for the biodegradation of high molecular weight polymers is oxidation or hydrolysis by enzymes to create functional groups that increase their hydrophilicity. The main polymer chains are degraded, producing polymers with a low molecular weight and feeble mechanical properties, which makes them more accessible for further microbial assimilation. Examples of synthetic polymers that biodegrade include poly(vinyl alcohol), poly(lactic acid), aliphatic polyesters, polycaprolactone, and polyamides. Several oligomeric structures are known to biodegrade: oligomeric ethylene, styrene, isoprene, butadiene, acrylonitrile, and acrylate.

Biodegradation is governed by different factors that include polymer characteristics, the type of organism, and the nature of pretreatment. Polymer characteristics play important roles in their degradation, including mobility, tacticity, crystallinity, molecular weight, the type of functional groups and substituents present in their structure, and the plasticizers or additives added to the polymers. Their physical properties also affect the rate of degradation, such as crystallinity, orientation, and morphological properties such as surface area [25].

PE is the most abundant synthetic, petroleum-based plastic material produced globally and one of the most resistant to biodegradation, which causes it to accumulate in massive quantities in landfills. The process of biodegradation of PE is defined by four stages: biodeterioration, biofragmentation, bioassimilation, and mineralization. The complete biodegradation of PE requires that the molar mass and molecular mass of the polymer be reduced by fragmentation into smaller molecules that are subsequently catabolized by microorganisms. However, most studies of what is usually called the biodegradation of PE by microorganisms have reported only biodeterioration, and a few have reported biofragmentation. Evidence for bioassimilation and mineralization has so far been lacking. Complete PE biodegradation may be defined as the oxidation of intact polymers to highly fragmented polymers, with corresponding decreases in molar mass (Mw) and molecular mass (Mn), followed by the subsequent conversion of the polymer fragments to CO_2_ and biomass (under aerobic conditions).

A clear definition of what constitutes true biodegradation of PE is lacking and there is a general misunderstanding of the biodegradation process. Biodegradation has been defined as the change in surface properties or the loss of mechanical strength, assimilation by microorganisms, degradation by enzymes, backbone chain breakage, and subsequent reduction in the average molecular weight of the polymers. Biodegradation is also defined as the conversion of materials into less complex intermediates or end products by solubilization, simple hydrolysis, or the action of biologically formed entities such as enzymes and other products of the organism. Polymer molecules may break down to produce fragments in this process, but not always, although the integrity of the material decreases in this type of process [26]. A key weakness of these definitions of ‘biodegradable’ is that they do not contain any information about the location, timescale, and extent of the decomposition process [27].

The implementation of high-throughput metagenomic sequencing to provide insights into the microbial community structures and the abundance of plastic degradation genes present in an MSW landfill site indicated that plastic waste is biodegraded in anaerobic conditions. Microbial richness was high, and *Thermobifida fusca*, *Pseudomonas mendocina*, and *Nocardia* sp. were present. Functional analysis revealed an abundance of genes coding for enzymes involved in the degradation of plastic waste, such as oxidoreductase, cutinase, lipase, alkane 1-monooxygenase, poly(ethylene terephthalate) hydrolase, mono (2-hydroxyethyl) terephthalate hydrolase, styrene monooxygenase, styrene monooxygenase reductase, etc. [28].

Plastics can undergo degradation through abiotic and/or biotic processes. The former is an essential first step that precedes the latter, as biodegradation mechanisms require an initial abiotic degradation process. This creates materials with diminished structural and mechanical integrities, creating particles with higher surface-area-to-volume ratios, which are amenable to microbial action [29]. Abiotic degradation pathways may be separated into two distinct types of processes which depend on the polymer type. More concisely, they depend on whether the polymer consists solely of a C–C backbone, as in PP, PS, PVC, and PE, or if heteroatoms are present in the backbone, as in PET and PU. In the first case, the process is initiated by a random photolytic cleavage of a C–H bond, while in polymeric materials containing heteroatoms, hydrolysis is usually the initiating step [30]. However, these mechanisms have been described in unadulterated materials, and polymers are rarely used in their pure form, and therefore rarely occur in the environment in this form. Consequently, the described mechanistic pathways of degradation may be incomplete, and products are released during (bio)degradation [31].

Using a lysimeter, Muenmee et al. [32] simulated an open waste dump in which waste plastics (HDPE/LDPE, PP, PS) were biodegraded. Methane oxidation conditions were imitated with a flow of synthetic biogas through the lysimeter. They observed that methanotrophs were the principal decomposers in plastic biodeterioration, and their abundance tended to increase, particularly that of type I/II methanotrophs (*Methylobacter* sp./*Methylocella* sp.). HDPE had the highest K constant, (0.128 y^−1^), and LDPE had the lowest (0.048 y^−1^). The K values correlated well with the rate of methane oxidation. Adamcová and Vaverková [33] provided information about the biodegradability in a municipal solid waste landfill of biodegradable/degradable materials advertised as 100% degradable or certified as compostable. They described a 12-month decomposition process in a municipal solid waste landfill. During this time, the PE with the additive and plastic waste labelled as 100% degradable had not decomposed, and no physical changes had occurred; however, their colour had changed slightly and the plastic waste exhibited minor surface changes. In contrast, cellulose filter paper fully biodegraded after eight months in the landfill conditions.

One indicator of the degradation of waste plastic during landfilling is the carbonyl index (CI), which is the average value of different infrared spectra [34]. The CI average of fresh PE is 0.53, which is higher than that of excavated PE. CI values decrease over time, with values of 0.46 and 0.41 for PE with <10 years and >10 years of landfilling, respectively. The slight decrease in the carbonyl index of the PE samples with >10 years of landfilling could indicate that some degradation has taken place. After initial degradation, the samples may degrade via chain scission, crosslinking, and CO_2_ release.

## 4. Potential Pathways for the Transfer of Plastic Debris from Landfills to the Environment

The possible pathways for plastic losses from landfills encompass environmental processes, including mechanisms such as wind, flooding, leaching, and runoff, and the influence of biota, including their removal by animals. Sanitary and well-managed engineered landfills have physical barriers to prevent losses in these ways, and their locations typically have environmental attributes that significantly reduce the losses of plastics from these landfills to the environment. Open dumps, in contrast, have negligible barriers to stop the losses of plastics to the environment, i.e., open dumps have neither fencing, to stop the movement of macroplastics via winds, runoff, or floods, nor liners to prevent microplastic movement via leachate [35].

The majority of MPs in groundwater come from landfills. For example, Bharath et al. [36] found that 90% of the MPs in groundwater were derived from buried plastics. Based on their morphological characteristics, these plastic particles were probably derived from the breakdown of daily plastic products. Both of the nearby landfill sites had 92% of the microplastics that were found in the groundwater, which were derived from the fragmentation of the buried plastics. The main source of MPs transfer from the landfill sites to the groundwater were surface runoff, effluent, open dumping, and open burning. The results of Kazour et al. [14] also indicate that landfills are not final sinks of plastics, but potential sources of microplastics. This can create serious environmental problems because microplastics are not listed as a pollutant in the landfill regulations of any country.

Landfill outputs include leachate and gases, which are sources of N/MPs. Landfill leachate is produced when water percolates through waste deposits, and it contains various pollutants. Leachate generated during landfilling contains microplastics because some personal care products used worldwide have incorporated plastic microbeads and other plastics in landfills are degraded over time.

## 5. Microplastics in Landfill Leachate

As landfill leachate acts as a source of MPs, it should be adequately managed to mitigate its potential adverse effects on the nearby environment. It is estimated that 0.2–1.0 m^3^ of leachate is produced per ton of waste landfilled each year in Europe [37]. About 52 million tons of municipal waste were landfilled in the EU in 2018, which would correspond to 10.4 Mm^3^ of leachate. Based on an estimate of 291 particles of MPs per liter per year [38], this would mean that around 3.03 billion of these particles were released in that year. Another example comes from 30 cities in China, in which 1.3–3.2 m^3^ of leachate accumulated per ton of waste over 100 years [39]. If these residues are not properly treated, not only wastewater effluents, but also a great quantity of microplastics eventually find their way into natural ecosystems, where they pose a serious risk to the environment [40].

MPs contain a large variety of chemical additives such as bisphenol A (BPA), phthalates, and polybrominated diphenyl ethers, which are used during raw plastic synthesis to improve plasticity. These additives are endocrine disruptors and thus may exhibit toxic effects upon release. The concentrations of such plasticizers in plastic debris on remote and urban beaches are as follows: BPA, up to 35 ng/g on remote beaches and up to 700 ng/g on urban beaches; polybrominated diphenyl ethers, between 0.1 and 400 ng/g on remote beaches and up to 9900 ng/g on urban beaches; and phthalates, up to 3940 ng/g [41]. These plastic additives have been detected in most microplastic polymers [42]. Researchers have also reported that BPA and nonylphenol leach from silicone and polycarbonate microplastics [43].

MPs in leachate can be carried into the environment through leachate leakage and the discharge of leachate by collecting and treatment systems. Leakage through landfill liners is a potential pathway for microplastics to enter the environment, and despite carefully controlled manufacturing and installation, defects in landfill liners (geomembranes) can occur. The concentration of MPs in leachate was reported as 0.42−24.58 items/L in southern China [44] and 0−4.51 items/L in Nordic countries [45]. In 88 Norwegian landfills in 2007, 9,100,000 m^3^ of leachate formed and 80 g of fine-grained plastics (PBD99) were emitted per year [46]. If this measured amount were recalculated as emissions of, for example, PBD-contaminated plastic dust concentrations such as those found in shredder dust (with a concentration of fine-grained plastics of 7.5 mg PBD 99 per kg), this would hypothetically equate to landfill emissions of (80.000/7.5) 10 tons of plastic particles annually. This is assuming that all the microplastics in the leachate were in particulate form. Additionally, 441 kg of BPA were found in the total leachate. This substance is typically present in polycarbonate plastics and epoxy plastics. According to industry sources, it is bound in the material, so no emissions should be expected. However, even though there was no evidence of the breakdown of these plastics in Norwegian landfills, the amount of BPA found in the leachate indicates that the plastics are not completely inert and may release free monomers or particles [46]. Kilponen [47] reported that, in a brook that received leachate from an old, closed landfill in Finland for over 30 years, MPs concentrations reached 1.1 items/L. MPs were found in landfill leachate in Nordic countries, including known polymers such as PE, PA, PVC, PET, PU, tire rubber, and polymer modified bitumen. Their levels did not seem to correspond to the period in which each landfill was operative. There was considerable variation both between the landfills and in the minimal and maximal loads of MPs. The approximate annual load of MPs emitted from the landfills in the particle size range of 50–5000 µm was 0–177 kg/landfill. Most particles were in the lower part of the size range, close to 50 µm, and their maximum loads were 25.3 kg per landfill [45].

Sun et al. [48] reported that the MPs particle and mass concentrations in leachate were 235.4 ± 17.1 item/L and 11.4 ± 0.8 μg/L, respectively. Over 50% of the MPs in leachate were less than 50 μm in size, and MPs with sizes > 100 μm and 50–100 μm accounted for 22% and 26%, respectively. The neutral buoyancy of the MPs (average density: 0.94 g/cm^3^) and their irregular shapes suggested they may be difficult to remove by sedimentation. In the leachate, over 90% of the MPs were transparent or yellowish, while some (<10%) were red, pink, purple, black, blue, and brown. The high abundance of transparent and yellowish colors suggested that most particles were aged and had been present in the landfill system for a long time.

He et al. [44] identified 17 different types of plastics in leachate samples with concentrations ranging from 0.42 to 24.58 items/L. PE and PP were the predominant polymer types. A total of 99.36% of the MPs were derived from the fragmentation of plastic waste buried in landfills. The size of 77.48% of the microplastics was between 100 to 1000 μm. According to Xu et al. [49], the abundance of target MPs in raw landfill leachate was 291 ± 91 particles/L. PP (40%), PA (36%), and rayon (18%) were the main types of MPs that were detected, while PP, polyester PET, and other kinds of MPs were also found occasionally. Approximately 90% of the MPs were ≤60 μm. Ignoring small MPs, especially those smaller than 50 μm, can cause bias when determining the abundance of MPs in landfill leachate samples. Su et al. [16] examined landfills with different ages (3~20 years) and found that the average abundance of MPs in leachate was 8 (±3) items/L. The predominant shapes and polymer types of the detected MPs were fibers and cellophane. The MPs in the landfill leachate had sizes that ranged from 3.6 to 70 μm.

MSW landfill sites are the destination for many types of chemical substances contained in the disposed MSW. Microcontaminants such as nonylphenol, phthalate acids, and BPA can leach from plastic materials after the weathering or aging of microplastic particles. Because of their hydrophobic nature and high surface area, they can interact with other pollutants that are present. Among these substances, BPA, a potential endocrine disrupting chemical, is frequently found in leachate from MSW landfills with a concentration that varies widely, from 1.3 to 17,200 µg/L. Yamamoto et al. [50] stated that the source of BPA in landfill leachate may be the waste plastics that were landfilled. Similarly, a study by Narevski et al. [51] reported that average BPA concentrations, which ranged from 0.70 to 2.72 mg/L, are related to the content of microplastics. When wastes containing BPA are disposed of in landfills, hydrolytic or leaching processes may occur, which can release BPA from these wastes to the leachate. It is well-known that the behaviour of organic contaminants in landfills is influenced by the combined effects of biodegradation, sorption, and transfer in the gas and/or liquid phases (i.e., volatilization and abiotic transformation, such as hydrolysis).

BPA is incorporated into plastic polymers through either reaction or addition. Reaction produces plastic products with covalently bonded stabilizers that are less likely to leave the products. In the addition process, compounds are simply mixed with the polymer resins, so they can continually leach out of these products. Another potential source of BPA in MSW landfill leachate is paper waste, which is usually neglected when BPA pollution is considered. In fact, paper waste can make up 32.7% of MSW [52]. For a preliminary insight into the source and leaching behaviour of BPA from MSW, Xu et al. [49] leached five kinds of plastic and four kinds of paper materials with distilled water. PVC waste was found to have the highest BPA content of 12.1 µg/g and a leachability of 34.7% in distilled water, while cardboard had a relatively low BPA content but also displayed high leachability (53.6%). Subsequently, those authors used fresh leachate and leachate from landfills aged 1.5 and 10 years, to simulate the leaching of BPA from PVC plastic and cardboard in a landfill environment. More leaching took place with the leachate than with distilled water. The 10-year-old leachate caused the greatest increase in leaching due to its basic pH and high content of humic organic matter. The fresh leachate increased BPA leaching more than the 1.5-year-old leachate, possibly due to the presence of small molecules such as volatile fatty acids, amino acids, etc., in the fresh leachate. The paper waste was only a minor source of BPA leaching, and interestingly, it was an important factor in retarding BPA transformation, due to its BPA sorption K_f_ value of 0.2224 mg ^(1 − n)^/(L^n^ g) (n = 0.7680; Freundlich equation), which is higher than that of natural organic adsorbents such as sediment. This finding suggests that the presence of paper with a high sorption capacity in MSW will reduce BPA transport and bioavailability in landfills.

## 6. Landfill Gases as a Source of Nano-/Microplastics

Small particles or fibers can be spread from landfills to the surrounding environments via the air. Indeed, the windborne movement of plastics to the nearby environment is a key challenge of operating landfills. Even though landfills have tried to reduce the scope of this problem and some cleanups have been performed, plastic waste around active and former landfills remains a source of MPs. For investigating the atmospheric deposition of MPs, living organisms (biomonitors) could be very useful, as they are in the monitoring of other important persistent air pollutants, but to date, studies on this topic are very scarce. Roblin and Aherne [53] evaluated the use of bryophytes (*Hylocomium splendens*) as biomonitors of airborne anthropogenic microfibers. Loppi et al. [54] explored the possibility of using lichens as bioaccumulators of airborne MPs. To investigate the amount of MPs that landfills release to the wider environment and the area over which they are released, they compared the accumulation of MPs in lichens at various distances from a landfill. Lichens from the vicinity of the landfill accumulated the highest number of anthropogenic microfibers and fragments (147 mp/g dw), and consequently, the largest number of microplastics (79 mp/g dw), suggesting that the impact of landfill emissions is spatially limited. The proportion of fibers and fragments identified as MPs was 40% across all sites, and the most abundant polymer type was polyester or PET (68%).

The aging of plastic debris in typical environmental conditions determines the release of harmful volatile organic compounds via oxidative photodegradation processes. Some compounds emitted are typical of the specific polymeric material. For example, Lomonaco et al. [23] found that aromatic compounds were produced only from polyolefins, and that PET had a negligible release profile which mainly included very modest quantities of ketones and aromatics. Most volatile compounds that are released play key roles in atmospheric chemistry because they are involved in complex photochemical reactions. For example, aldehydes are prone to atmospheric photolysis, contributing to the formation of airborne particles; similarly, the photooxidation of benzene, toluene, ethylbenzene, and xylene produces important precursors of secondary organic aerosols [55]. Royer et al. [56] demonstrated that environmentally aged LDPE, incubated for 14 days at ambient outdoor temperature (18.5–32.5 °C), produced 0.37 ± 0.11, 0.14 ± 0.09, 0.21 ± 0.12, and 0.06 ± 0.03 nmol/g per day of CH_4_, C_2_H_4_, C_2_H_6_, and C_3_H_6_, respectively. They reported that, as the surface-to-volume ratio increased, so did the rate at which these compounds were produced, indicating that they may be released throughout the lifetime of the plastics as long as they remain exposed to thermal and/or photooxidative degradation conditions.

## 7. Environmental Effects of Plastics and MPs

The adverse effects of MPs exposure on organisms can be divided into two categories: physical effects and chemical effects. The former is related to the size and shape of the particles, and the concentration of the MPs, whereas the latter is related to the hazardous chemicals that are released from the MPs. MPs can contain two types of chemicals: (1) additives and polymeric raw materials (e.g., monomers or oligomers) originating from the plastics; and (2) chemicals absorbed from the surrounding environment. These include inert or reinforcing fillers, plasticizers, antioxidants, UV stabilizers, lubricants, dyes, and flame retardants. Among the additives, wood and rock flour, clay, kaolin, graphite, glass fibers, cotton flakes, jute or linen, cellulose pulp, etc., are used. The additives, in almost all cases, are not chemically bound to the plastic polymer; only some flame retardants are polymerized with plastic molecules, becoming part of the polymeric chain. Though these additives improve the properties of polymeric products, many of them are toxic, and their potential to contaminate the soil, air, and water is high [57]. MPs have a high surface-area-to-volume ratio, making them a good sorbent for toxic chemicals, such as heavy metals and organic chemicals.

The transport and deposition of chemical contaminants by MPs can vary depending on the age, concentration, and type of the contaminant (e.g., additives, heavy metals, persistent organic contaminants, antibiotics, pesticides, fungicides). Organic contaminants are most commonly adsorbed on microplastics via hydrophobic interactions, followed by electrostatic interactions, H-bonding, halogen bonding, and π–π interactions. Metal ions are adsorbed mainly by electrostatic interactions and surface complexation. For both organic and inorganic contaminants, the adsorption kinetics data are best described with a pseudo-second-order (PSO) model, but depending on the system, the isothermal data are best described by either the Freundlich or the Langmuir isotherm model [58]. Holmes et al. [59] found that heavy metals displayed a greater affinity for PVC and PP than other MPs. The ranges of metal concentrations adsorbed on PE resin pellets decreased in the following order: Cr (44–430 μg/g) > Ni (40–131 μg/g) > Fe (41–97.8 μg/g) > Co (17.7–107 μg/g) > Cd (1.09–76.7 μg/g) > Al (16.9–55.8 μg/g) > Zn (0.299–23.3 μg/g) > Mn (1.16–20.5 μg/g) > Cu (0.064–1.32 μg/g). Munier and Bendell [60] reported the following ranges of mean metal concentrations on different MP surfaces: Cd, 0.37 μg/g on PP to 1.77 μg/g on LDPE; Cu, 2.93 μg/g on nylon to 47.53 μg/g on LDPE; Zn, 4.3 μg/g on PVC to 604.24 μg/g on LDPE; and Pb, 0.71 μg/g on PET to 17.68 μg/g on LDPE. In general, LDPE had larger amounts of metals adsorbed to its surface. The large loads of toxic metals on the surface of MPs indicates that they can serve as alternative sources or sinks of toxic metal contaminants in the environment. However, data on toxic metal adsorption onto MPs that are present in the soil or air are limited.

MPs convey contaminants to organisms and between environment media. Liu et al. [61] investigated the sorption behaviour of two phthalate esters (PAEs), diethyl phthalate (DEP) and dibutyl phthalate (DBP), on three types of microplastics with particle sizes less than 75μm: PVC, PE, and PS. The amount of the two PAEs that sorbed on the three microplastics decreased in the following order: PS > PE > PVC. With each kind of MP, DBP sorption was almost 100 times greater than that of DEP, demonstrating the predominant influence of hydrophobic interactions on partition.

The sorption capacity of MPs is influenced by their physicochemical properties. The surface charge of MPs, which is influenced by the presence of functional groups in their structure, can affect the adsorption of polar compounds such as pharmaceuticals. For example, Elizalde-Velazquez et al. [62] found that PE shows a great sorption affinity for pharmaceuticals, such as nonsteroidal anti-inflammatory drugs (NSAIDs). Polyamide has a porous structure and can form hydrogen bonds with antibiotics. Adsorption isotherms demonstrated that polyamide had the highest capacity for adsorbing antibiotics with distribution coefficient (Kd) values ranging from 7.36 ± 0.257 to 756 ± 48.0 L/kg. Li et. al. [63] reported that the amounts of antibiotics adsorbed on PS, PE, PP, and PVC decreased in this order: ciprofloxacin > amoxicillin > trimethoprim > sulfadiazine > tetracycline. Smaller MPs have a higher specific surface area, and thus provide more adsorption sites and have a higher sorption capacity. This was confirmed by Elizalde-Velazquez et al. [62], who found that a decrease in the particle size led to an increase in the amount of NSAIDs sorbed to MPs.

When antibiotics are adsorbed on MPs, they can be transported over long distances, and may contribute to compound effects by interacting with other substances that have been absorbed. Imran et al. [64] concluded that co-contamination with MPs, metals, and antibiotics results in the development and spread of multiple drug-resistant human pathogens through a co-selection mechanism. MPs act as hotspots for the metal-driven co-selection of multidrug-resistant human pathogens. Wu et al. [65] quantified antibiotics, heavy metals, and 28 antimicrobial resistance genes (ARGs) in refuse and leachate from landfills of different ages (<3, >10, >20, and >29 years). They found that the drivers and patterns of antibiotic release differ as a function of landfill ages. Specifically, younger landfills may release higher levels of antibiotics than older landfills, presumably due to degradation or changes in usage patterns. Shi et al. [66] indicated that municipal landfill leachate is a huge reservoir of N/MPs and ARGs. N/MPs have a proven ability to affect the growth of bacteria and the composition of bacterial communities, which may further influence the spread of ARGs in the environment. The presence of N/MPs in landfill leachate can facilitate the propagation of ARGs, which is closely linked to N/MPs effects on cellular membrane permeability. The long-term exposure of leachate to MPs having a size of 200–500 nm was proved to promote the propagation of ARGs, due to the MPs large effects on the composition of ARG-hosting bacteria and the great damage they cause to bacterial membranes. The enrichment of ARGs induced by N/MPs substantiates the urgent need for the strict management of plastic waste in MSW landfills to control the spread of ARGs [66].

The aging process affects the behaviour of MPs in the environment by increasing their specific surface area, the roughness of their surface morphology, and the formation of hydrophilic groups, which increases the likelihood of microbial colonization [67]. The long-term aging of MPs in effluent changes their properties and increases their potential to serve as vectors for ARGs. Bacterial consortiums on MPs display a greater potential for biofilm formation and pathogenicity than consortiums in the effluent. Moreover, MPs appear to selectively enrich ARGs, and the aging process seems to increase the enrichment potential. Finally, it is important to keep in mind that ARGs are present not only in the bacteria, but also in the extracellular DNA in the biofilms that they create on the surface of MPs, and this extracellular DNA is an important reservoir of ARGs.

## 8. Conclusions

Plastics persist and pollute long after their intended use is finished, which means that there is no such thing as an end to the impact of plastics within a typical human lifespan. Depending on how plastic is handled, it may pose a significant threat to the environment and to the climate when it reaches the waste phase of its life cycle. The direct impact of landfills on the formation of MPs in the environment is due to the way landfills are operated, the impact of environmental processes such as wind, flooding, leaching, and runoff, and the distribution of waste by animals. Landfills have tried to reduce the scope of this problem, but plastic waste around active and former landfills remains a source of MPs. The indirect impact of landfills on the formation and spread of MPs is due to the structure of plastic waste containing nano-/microparticles and the defragmentation of plastic waste during landfilling. Fine particles or fibers can be transported from landfills to the surrounding environment by leachate and air. The aging of plastic debris in typical environmental conditions determines the release of harmful volatile organic compounds via oxidative photodegradation processes. In addition, MPs act as carriers or chelators of various co-contaminants, such as heavy metals, brominated flame retardants and other types of plasticizers, and pharmaceutical toxicants. These co-contaminants easily bind to the microplastic surface due to their hydrophobicity. MPs contain a large variety of chemical additives such as bisphenol A (BPA), phthalates, and polybrominated diphenyl ethers, which are used during raw plastic synthesis to improve plasticity. These additives are endocrine disruptors, and thus may exhibit toxic effects upon release. It is necessary to generate, analyze, and disseminate an ever more extensive dataset on landfills that includes information on the characteristics and variability of MPs, including changes over time, which will necessitate multi-annual studies.

To understand MPs degradation patterns and the most common types of polymers that they contain, it is necessary to characterize MPs from landfills. This will help to develop an understanding of the environmental effects of MPs from landfills. To better understand the indirect effects of MPs pollution, it will be necessary to perform holistic multidisciplinary studies that address the migration and fate of MPs, as well as their role in the dissemination and evolution of antibiotic resistance. The elements of plastic waste that are most prone to fragmentation that could be reduced by improved product design strategies should be identified, as well as methods of reducing consumption or increasing recycling. The landfilling of plastics should be avoided by implementing strategies to prioritize the reduction, recycling, and conversion of waste into energy, and by campaigning to change consumer behaviour, in particular by avoiding the purchase of short-lived products, such as single-use plastics.

## Data Availability

Not applicable.
